# Differential effect of DJ-1/PARK7 on development of natural and induced regulatory T cells

**DOI:** 10.1038/srep17723

**Published:** 2015-12-04

**Authors:** Yogesh Singh, Hong Chen, Yuetao Zhou, Michael Föller, Tak W. Mak, Madhuri S. Salker, Florian Lang

**Affiliations:** 1 Department of Physiology I, Eberhard-Karls-University of Tübingen, Tübingen, D-72076, Germany; 2 Campbell Family Institute for Breast Cancer Research, Ontario Cancer Institute, UHN, 620 University Ave Toronto, M5G 2C1, Canada

## Abstract

Regulatory T cells (Tregs) are essential for maintaining an effective immune tolerance and a homeostatic balance of various other immune cells. To manipulate the immune response during infections and autoimmune disorders, it is essential to know which genes or key molecules are involved in the development of Tregs. Transcription factor Foxp3 is required for the development of Tregs and governs most of the suppressive functions of these cells. Inhibited PI3K/AKT/mTOR signalling is critical for Foxp3 stability. Previous studies have suggested that DJ-1 or PARK7 protein is a positive regulator of the PI3K/AKT/mTOR pathway by negatively regulating the activity of PTEN. Thus, we hypothesised that a lack of DJ-1 could promote the development of Tregs. As a result, loss of DJ-1 decreased the total CD4^+^ T cell numbers but increased the fraction of thymic and peripheral nTregs. In contrast, Foxp3 generation was not augmented following differentiation of DJ-1-deficient naïve CD4^+^ T cells. DJ-1-deficient-iTregs were imperfect in replication, proliferation and more prone to cell death. Furthermore, DJ-1 deficient iTregs were less sensitive to pSmad2 and pStat5 signalling but had activated AKT/mTOR signalling. These observations reveal an unexpected differential role of DJ-1 in the development of nTregs and iTregs.

Regulatory T cells (Tregs) are pivotal for maintenance of thymic and peripheral self-tolerance and protection against unwanted collateral damage induced during inflammation^1^,^2^. In addition, these cells are similarly induced in bacterial infections such as Mycobacterium tuberculosis and debilitate the host to combat the infection^3^. Tregs further promote the growth of cancer^4^,^5^. Tregs not only modulate adaptive immunity but are also involved in the modulation of innate immunity^6^. Adequate development and function of Tregs is thus vital to maintain the homeostatic balance and overcome pathological conditions. Accordingly, Tregs are an attractive immunotherapeutic target^7^.

Tregs are generated in the thymus designated as natural Tregs (nTregs) and in the peripheral organs by antigen driven conversion of naïve CD4^+^ T cells in the presence of TGF-β and IL-2 considered as induced Tregs (iTregs)^8^. The Fork-head box p3 (Foxp3) transcription factor governs most of the functions and developmental pathways of both thymic and peripheral Tregs. Induction of Foxp3 in peripheral naïve T cells is controlled by various pathways such as Phosphatidylinositide 3-kinase (PI3K)/AKT/mTOR and TGF-β/SMAD2/3^9^,^10^. The proteins involved in these pathways are instrumental in modulating the functions and development of Tregs. Potential regulators of PI3K-dependent signalling include Parkinsons Disease Protein 7 (PARK7) or Oncogene DJ-1 (PARK7 or DJ-1), which negatively regulate the Phosphatase and tensin homolog (PTEN) acivity^11^,^12^ thus leading to activation of the PI3K pathway. Increased activity of PI3K-dependent AKT/mTOR signalling fosters cell growth and proliferation and downregulates Foxp3 protein, a transcription factor decisive in the development of Tregs (Tregs)^13^,^14^.

DJ-1 or PARK7 is ubiquitously expressed in human tissues and might play an important role in post-transcriptional gene expression^15^,^16^. Furthermore, DJ-1 acts as a positive transcriptional co-regulator of the androgen receptor (AR) by preventing protein inhibitor of activated STAT (PIASxα)/androgen receptor interacting protein 3 (ARIP3) and DJ-1 binding protein (DJBP)^17^. This protein is a redox-sensitive chaperone and protects neurons and the heart against oxidative stress and cell death^15^,^18^. Deletion of the DJ-1 gene exacerbates the progression of Parkinson´s disease^19^. As DJ-1 protein is also involved in the regulation of cell survival through AKT/mTOR, it protects cancer cells against hypoxia-induced cell death and is required for adaptation to hypoxic stress^12^. In cardiomyocytes, the DJ-1/PTEN/AKT pathway similarly protects against oxidative stress^18^,^20^,^21^. Oxidative stress engaged defences induce transient, mild mitochondrial depolarization^22^. Recent observations suggest that DJ-1 protein regulates CD3^+^ T cell migration through controlling the expression of the CXCR4 receptor^23^. However, to the best of our knowledge, an impact of the DJ-1 protein in the CD4^+^CD25^+^Foxp3^+^ regulatory T cell development has not been reported.

Here, we show (based on Foxp3 flow staining) that DJ-1 deficiency leads to enhanced percentage of thymic as well as peripheral nTregs in the CD4^+^ T cells compartment. DJ-1 deficiency further modifies the CD4^+^/CD8^+^ T cell ratio. DJ-1 deficient mice have lower total number of CD4^+^ T cells compared to control littermates in both thymus and spleen. However, when naïve T cells are differentiated into induced Tregs (iTregs) using TGF-β and IL-2, Foxp3 induction was significantly attenuated in DJ-1 deficient mice, which was contrary to naturally occurring peripheral Tregs development. Functions of nTregs from both mice strains were not impaired. Furthermore, DJ-1 deficient iTregs also had defective cell cycle progression and were more prone to cell death compared to the respective WT cells. Additionally, iTregs from DJ-1 mice were less sensitive towards phosphorylation of Smad2 and Stat5 proteins and had higher AKT phosphorylation and Rictor/mTOR activity, which could lead to impaired development. Thus, our data reveals a unique role of DJ-1 in iTregs development.

## Results

### DJ-1 deficiency augments the percentage of nTregs both in the thymus and periphery, but decreases the total CD4^+^ T cell numbers

**Figure 1:**
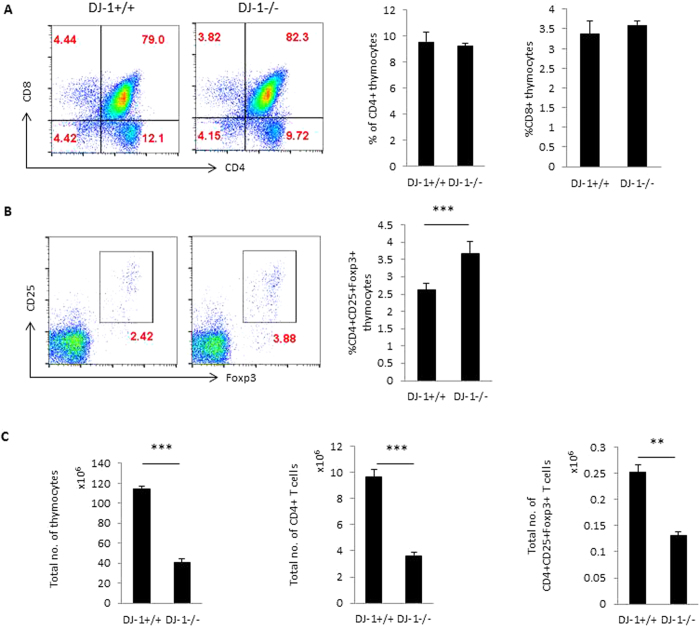
Thymic nTregs development in DJ-1 deficient mice. (**A**) DJ-1^+/+^ and DJ-1^−/−^ thymocytes were stained with anti-CD4 and anti-CD8 mAbs and characterised for CD4 and CD8 expression. No significant difference was observed between DJ-1^+/+^ and DJ-1^−/−^ in CD4^+^ or CD8^+^ thymocytes. Left hand side shows the representative FACS plots for CD4 and CD8 staining and right hand side shows the respective mean ± SEM (n = 3–5 independent experiments). (**B**) Thymocytes were stained with CD4, CD8, CD25 and Foxp3 antibodies and thymocytes were gated on CD4^+^ thymocytes. CD4^+^ thymocytes were further characterised for CD25 and Foxp3 expression. As a result, CD4^+^CD25^+^Foxp3^+^Tregs are significantly more abundant in DJ-1^−/−^ CD4^+^ thymocytes than in DJ-1^+/+^ CD4^+^ thymocytes (p = 0.03). Left hand side shows the representative FACS plots for CD25 and Foxp3 staining gated on CD4^+^ thymocytes and right hand side mean ± SEM (n = 3–5 independent experiments) (**C**) Total thymocytes were counted and estimated total CD4^+^ T cells and CD4^+^CD25^+^Foxp3^+^ thymocytes, data derived from 5 biological replicates.

**Figure 2:**
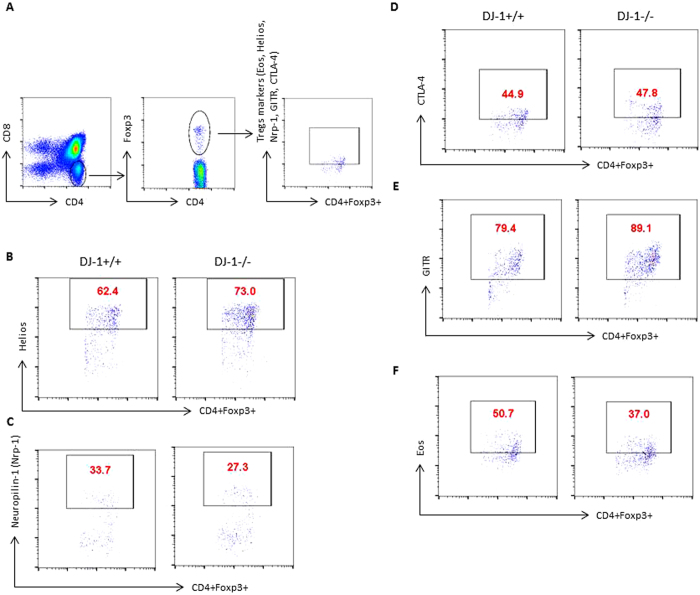
Characterisation of thymic nTregs in DJ-1 deficient mice. (**A–E**) DJ-1^+/+^ and DJ-1^−/−^ thymocytes were stained with anti-CD4 and anti-CD8 mAbs and characterised for Foxp3, Eos, Helios, CTLA-4, GITR and Nrp-1 expression. FACS plots represent the staining for total thymocytes for CD4^+^Foxp3^+^ *versus* Helios, Nrp-1, CTLA-4, GITR and Eos. (n = 2 independent experiments and 5–6 biological replicates/group). (Mean ± SEM of all FACS plot shown in Suppl. Fig. 1).

To examine the role of DJ-1 protein in T cell development, we first characterised the development of T cells in thymocytes from DJ-1^−/−^ mice. The development of T cell receptors (TCRs) in the thymus was similar in both DJ-1^−/−^ and DJ-1^+/+^ mice in the pre-TCR stage before CD4^+^ and CD8^+^ thymocyte maturation (data not shown). No significant change was also observed of single positive CD4 or CD8 thymocyte development in both strains of mice (Fig. 1). However, CD4^+^CD25^+^Foxp3^+^ thymocytes (thymic derived natural Tregs or nTregs) were significantly more abundant in DJ-1^−/−^ mice than in DJ-1^+/+^ mice as measured by flow cytometry at percentage level (Fig. 1B). Further analysis revealed that due to less thymic cellularity the total number of CD4^+^ thymocytes and nTregs were significantly reduced in DJ-1^−/−^ mice compared with control (DJ-1^+/+^) littermates (Fig. 1C). In search for any defect in expression of various other key Tregs markers, we characterised the nTregs using several known markers such as Helios (Ikzf2), Eos, CTLA-4, GITR, Neuropilin-1 (Nrp-1) in addition to CD25 and Foxp3. As a result, the percentage of DJ-1^−/−^ nTregs had a significantly higher expression level of Helios, GITR and significantly lower expression of Nrp-1. No significant change was observed in CTLA-4 and Eos in both strains of mice (Fig. 2 and Suppl. Fig. 1). Again due to a reduced thymic cellularity, total numbers of various marker positive cells were significantly reduced in DJ-1^−/−^ nTregs compared to control group except for CTLA-4 marker (Fig. 2 and Suppl. Fig. 1).

**Figure 3:**
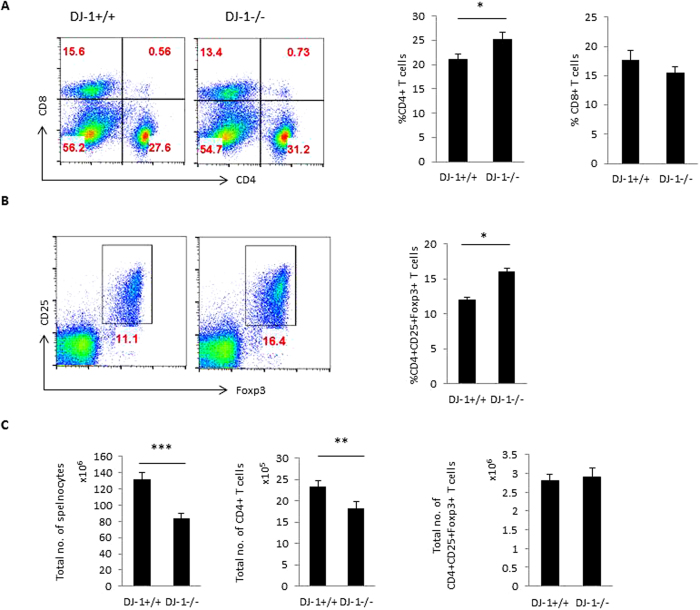
Peripheral development of Tregs (pTregs) in DJ-1 deficient mice. (**A**) DJ-1^+/+^ and DJ-1^−/−^ splenocytes were stained with anti-CD4 and anti-CD8 mAbs and characterised for CD4 and CD8 expression. Difference between DJ-1^+/+^ and DJ-1^−/−^ mice CD4^+^ T cells splenocytes were subtle but statistically significant (p = 0.036), however, no significant difference was observed between DJ-1^+/+^ and DJ-1^−/−^ CD8^+^ T splenocytes. Left hand side shows the representative FACS plots for CD4 and CD8 staining and right hand side shows the mean ± SEM (n = 5–7 independent experiments). Data were obtained from 11 biological replicates. (**B**) Splenocytes were stained with CD4, CD8, CD25 and Foxp3 antibodies and splenocytes were gated on CD4^+^ T cells. CD4^+^ T cells were characterised for Tregs markers CD25 and Foxp3. As a result, CD25^+^Foxp3^+^Tregs are more abundant in DJ-1^−/−^ mice than in DJ-1^+/+^ mice (p = 0.011). Left hand side shows the representative FACS plots for CD25 and Foxp3 staining gated on CD4^+^ T cells and right hand side shows mean ± SEM (n = 4 independent experiments). (**C**) Total splenocytes were counted and estimated CD4^+^ T cells as well as CD4^+^CD25^+^Foxp3^+^ T cells numbers, data were derived from 5 biological replicates.

**Figure 4:**
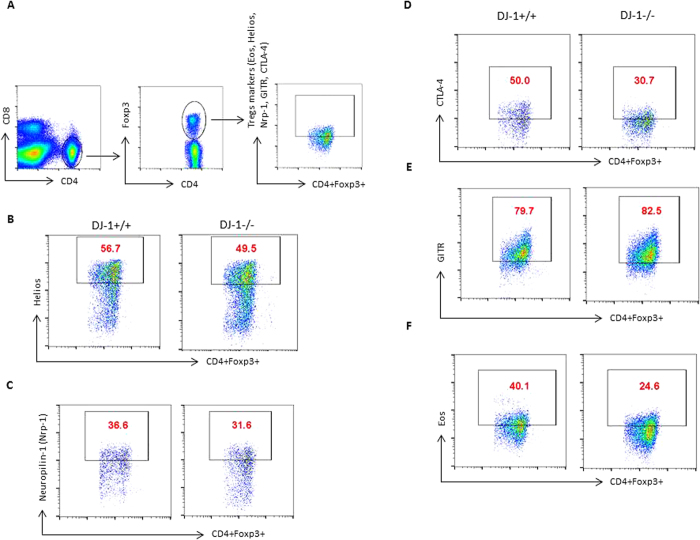
Characterisation of nTregs in DJ-1 deficient mice from iTregs. (**A–E**) DJ-1^+/+^ and DJ-1^−/−^ splenocytes were stained with anti-CD4 and anti-CD8 mAbs and characterised for Foxp3, Eos, Helios, CTLA-4, GITR and Nrp-1 expression. FACS plots represent the staining for CD4^+^ T cells gated for CD4^+^Foxp3^+^ *versus* Helios, Nrp-1, CTLA-4, GITR and Eos. (n = 2 independent experiments and 5–6 biological replicates/group). (Mean ± SEM of all FACS plot shown in Suppl. Fig. 2).

Next, we examined the peripheral development of CD4^+^ T cells and CD4^+^CD25^+^Foxp3^+^ regulatory T cells (pTregs) in DJ-1^−/−^ mice. The percentage of CD4^+^ T cells were significantly higher in DJ-1^−/−^ mice than in DJ-1^+/+^ mice (Fig. 3A). CD8^+^ T cells tended to be lower in DJ-1^−/−^ mice than in DJ-1^+/+^ mice, a difference, however, not showing statistical significance (Fig. 3A). The percentage of pTregs was significantly higher in DJ-1^−/−^ mice than in DJ-1^+/+^ mice (Fig. 3B). Due to less splenic cellularity in DJ-1 deficient mice, the total number of CD4^+^ T cells was significantly reduced. However, no significant change was observed in total pTregs cell number (Fig. 3C). The pTregs compartment is made of nTregs and iTregs expressing the canonical Tregs markers such as CD25, Foxp3, GITR and CTLA-4, but nTregs express more Nrp-1, Helios, programmed cell death-1 (PD-1) and CD73 than iTregs^24^,^25^. We stained the splenic cells with Helios and Nrp-1, which was used to differentiate between nTregs from iTregs as described previously^25^,^26^. Based on flow staining, no significant change was observed in Helios and Nrp-1 expression in both strains of mice in CD4^+^Foxp3^+^Helios^+^ and CD4^+^Foxp3^+^Nrp-1^+^ Tregs (Fig. 4 and Suppl. Fig. 2). Furthermore, CTLA-4 expression was significantly lower in DJ-1^−/−^ mice than in DJ-1^+/+^ mice in CD4^+^Foxp3^+^CTLA-4^+^ Tregs; no change was observed in GITR expression in CD4^+^Foxp3^+^GITR^+^ Tregs in both strains of mice (Fig. 4 and Suppl. Fig. 2). In addition, we also characterised pTregs for Eos expression, a Tregs specific transcription factor that interacts with Foxp3 and is required for adequate suppressive functions in Tregs^27^,^28^. Here, we found that there was a significantly lower expression of CD4^+^Foxp3^+^Eos^+^ T cells from DJ-1 deficient mice compared to control littermates (Fig. 4 and Suppl. Fig. 2). DJ-1^−/−^ mice tended to have fewer splenic CD4^+^CD44^+^CD62L^-^ memory T cells and more splenic CD4^+^CD62L^+^CD44^-^ T cells than DJ-1^+/+^ mice, a difference, however, not reaching statistical significance (Suppl. Fig. 3A,B and C). In conclusion, both the thymic and peripheral splenic data suggest that DJ-1^−/−^ mice have a lowest total number of CD4^+^ T cells but a higher percentage of thymic and peripheral nTregs than DJ-1^+/+^ mice.

### DJ-1 deficient Tregs have no suppressive functional defect *in vitro*

**Figure 5:**
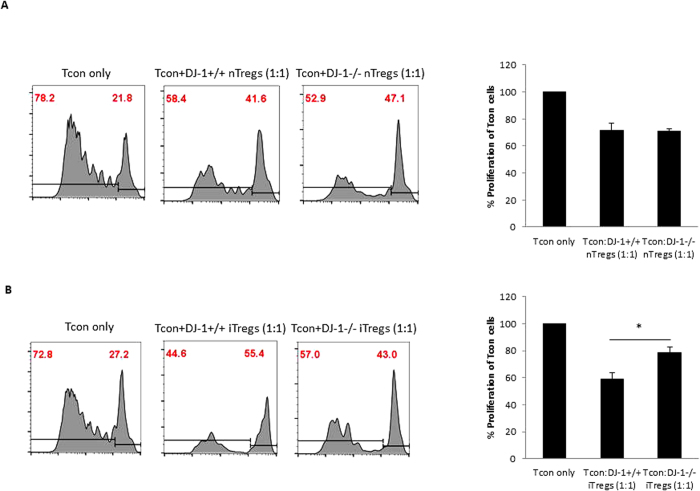
nTregs from DJ-1 deficient mice have no difference in suppressive in function. (**A**) DJ1^−/−^ and DJ-1^+/+^ nTregs were equally suppressive in functions as measured by co-culture experiments by using cell proliferation CFSE dye at day 4 of culture. The histogram represents cell proliferation of Tcon cells alone stained with CFSE (left) and co-cultured with DJ-1^+/+^ nTregs (middle) and co-cultured with DJ-1^−/−^ nTregs (right). (**B**) DJ1^−/−^ iTregs were less suppressive in functions as measured by co-culture experiments by using cell proliferation CFSE dye at day 4 of culture compared to DJ-1^+/+^ iTregs. The histogram represents cell proliferation of Tcon cells alone stained with CFSE (left) and co-cultured with DJ-1^+/+^ iTregs (middle) and co-cultured with DJ-1^−/−^ iTregs (right). Data are derived from 2 independent experiments (6 mice/group).

To explore the potential of nTregs to suppress conventional T cells (Tcon), we isolated CD4^+^CD25^+^ nTregs and performed a cell suppression assay using a CFSE dye to measure proliferative T cells in classical conventional co-culture experiments (Tcon:nTregs: 1:1). Proliferation of Tcon cells was inhibited to a similar extent by DJ-1^−/−^ and DJ-1^+/+^ nTregs (Fig. 5A). This result suggested that DJ-1^−/−^ and DJ-1^+/+^ nTregs are equally capable in suppressing the proliferation of other immune cells. We further examined the suppressive potential of iTregs derived *in vitro* from DJ-1^−/−^ and DJ-1^+/+^ mice and found that the suppressive capabilities when co-cultured into 1:1 ratio (Tcon:iTregs) was significantly impaired in DJ-1^−/−^ cells (Fig. 5B). However, enhanced proliferation in co-cultured experiments of Tcon:DJ-1^−/−^ iTregs compared with Tcon:DJ-1^+/+^ iTregs could be due to lowest total numbers of iTregs present in *in vitro* differentiated iTregs. Thus, these data suggest that DJ-1^−/−^ T cells differentiate less into iTregs compared to DJ-1^+/+^ T cells and did not accurately reflect measurement of suppressive capabilities of iTregs. Thus, this data confirm the lower iTregs induction in DJ-1^−/−^ compared with DJ-1^+/+^ mice.

### In DJ-1 deficient mice iTregs induction, cell cycle progression, cell survival and proliferation are compromised

**Figure 6:**
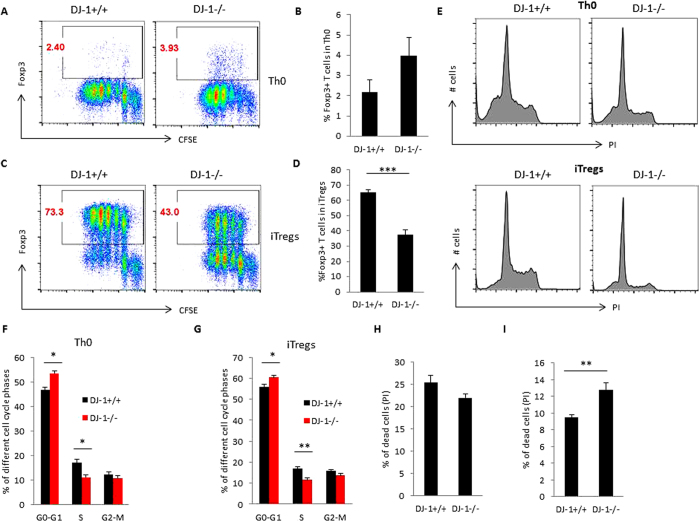
DJ-1 deficient mice have defective induction of iTregs, cell cycle progression and are more prone to cell death. (**A–D**) Purified CD4^+^ T cells were stained with the cell proliferation dye CFSE (2 μM) and cultured for 3 days with TGF-β (5.0 ng/ml) and IL-2 (10.0 ng/ml) (iTregs) and without both cytokines (Th0). After 3 days of culture cells were stained with a Foxp3 antibody and acquired by flow cytometry. Flow data suggested DJ-1^−/−^ mice have significantly fewer iTregs compared with DJ-1^+/+^ mice (p = 0.02). Left hand side shows the representative FACS plots for Foxp3 and CFSE staining and right hand side shows mean ± SEM (n = 3–5 independent experiments) for Th0 cells and iTregs. (**E–G**) Cell proliferation of Th0 and iTregs was characterised by Propidium Iodide (PI) staining. PI staining suggested DJ-1^−/−^ Th0 cells have significantly more G0-G1 cell cycle stages (p = 0.02) and fewer S phase stages compared with DJ-1^+/+^ Th0 cells and significance difference was observed at percentage level. Similar finding were also observed in iTregs from DJ-1^−/−^ mice: iTregs have significantly more G0-G1 cell cycle stages (p = 0.03) and significantly fewer S phase (p = 0.009) stages compared with DJ-1^+/+^ iTregs at percentage level. Data are shown as mean ± SEM (n = 5–6 independent experiments performed in duplicate). (**H,I**) Cell death analysis suggested DJ-1^−/−^ Th0 cells have no difference in cell death compared with DJ-1^+/+^ Th0 cells whereas DJ-1^−/−^ iTregs were significantly more prone (p = 0.002) to cell death compared with DJ-1^+/+^ iTregs. Data are shown as mean ± SEM (n = 5–7 independent experiments performed in duplicate).

DJ-1 is a negative regulator of PTEN and thus upregulates AKT and mTOR^12^,^20^, which have in turn been shown to inhibit the induction of Foxp3 in developing iTregs^10^,^13^,^14^. We thus presumed that deficiency of DJ-1 may affect the induction of Foxp3 in T cells and explored further whether deficiency of DJ-1 influences the induction of Foxp3 in developing iTregs. When we differentiated naïve CD4^+^ T cells into iTregs using TGF-β and IL-2, surprisingly, we found that instead of an increase in the induction of Foxp3, there was significantly reduced expression of Foxp3 in developing iTregs (Fig. 6A–D). However, naïve CD4^+^ T cells were left stimulated in the presence of anti-CD3 and anti-CD28 (Th0 cells), and the Foxp3^+^ cell number was higher in DJ-1^−/−^ mice than in DJ-1^+/+^ mice (Fig. 6A,B). These data suggested that *in vitro* DJ-1^−/−^ mice induce less Foxp3 in developing iTregs. To explore the possible cause of reduced induction of Tregs, we examined the cell cycle of Th0 and iTregs in DJ-1^−/−^ mice by Propidium Iodide (PI). PI staining suggested that DJ-1 Th0 cells had a significantly more percentage of G0-G1 and less percentage of S phase but no change in G2-M phase of the cell cycle (Fig. 6E,F) and similarly iTregs also had a significantly more percentage of G0-G1 and less percentage of S phase of the cell cycle (Fig. 6E,G). These results point to a proliferation defect in the absence of DJ-1 protein. However, it does not explain why DJ-1^−/−^ mice have less Foxp3 induction. Further, we checked the cell death analysis in iTregs and found that DJ-1^−/−^ developing iTregs were more prone to cell death compared with DJ-1^+/+^ developing iTregs (Fig. 6I). In contrast, no difference between the genotypes was observed in Th0 cells (Fig. 6H). Furthermore, Foxp3^−^ iTregs were significantly more proliferative in DJ-1^−/−^ compared with DJ-1^+/+^ mice, whereas no statistical difference was observed in Th0 cells between DJ-1^−/−^ and DJ-1^+/+^ mice (Suppl. Fig. 4). These data suggested that *in vitro* DJ-1^−/−^ mice have reduced cell cycle progression and more cell death in developing iTregs.

### AKT/mTOR signalling is activated whereas TGF-β/IL-2 signalling is less responsive in DJ-1 deficient iTregs

**Figure 7:**
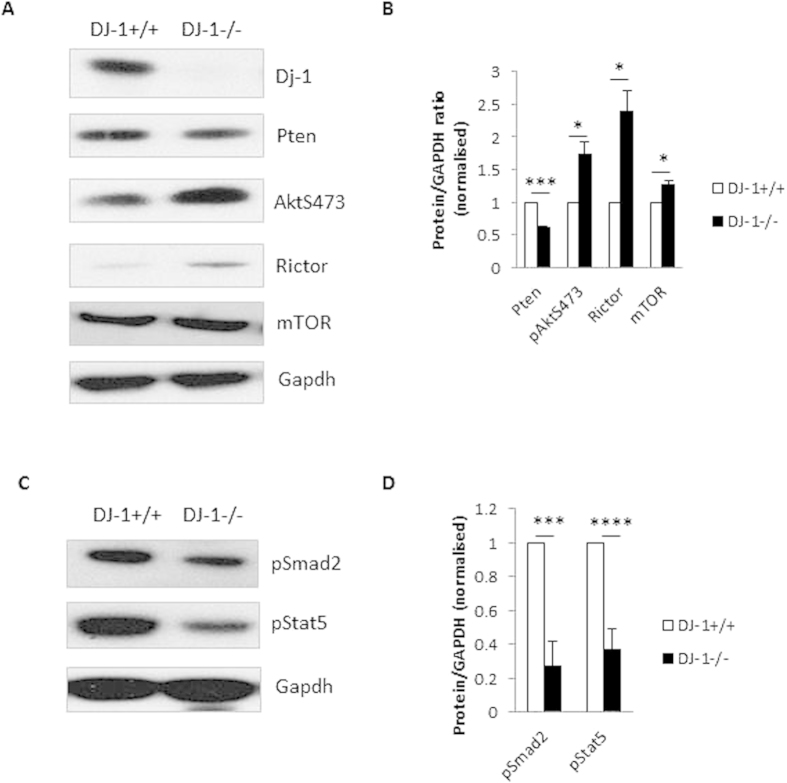
DJ-1 deficiency enhances the AKT/mTOR signalling pathways *via* TCR activation in iTregs, however less sensitive to TGF-β and IL-2 receptor signalling. (**A,B**) DJ1^−/−^ iTregs have significantly higher phosphorylation of AKT and expression of mTOR and Rictor proteins compared with DJ-1^+/+^ iTregs. Right hand bar diagram showed the statistical summary data derived from 5 mice/group. (**C,D**) TGF-β and IL-2 receptor signalling pathways proteins such as Smad2 and Stat5 have significantly lower phosphorylation activity in DJ-1^−/−^ compared with DJ-1^+/+^ iTregs. Right hand bar diagram showed the statistical summary data derived from 5 mice/group. Data are shown as mean ± SEM from 5 biological replicate mice (n = 2).

In the preceding section, we have shown that DJ-1 deficiency compromises the production of iTregs. It is known that DJ-1 also modifies the PTEN/mTOR/Rictor^10^, which is activated by TCR and CD28 signalling. Therefore, we explored the signalling pathways in iTregs. To our surprise, we found that PTEN expression was lower in iTregs from DJ-1^−/−^ than in iTregs from DJ-1^+/+^ mice, an observation in sharp contrast to other cell types^11^,^20^ (Fig. 7A). To confirm whether we had complete knock-down in DJ-1 deficient iTregs, we performed Western blotting against DJ-1 (Fig. 7A). In addition, both mTOR and Rictor were significantly higher in DJ-1^−/−^ than in DJ-1^+/+^ iTregs (Fig. 7A). As a result of higher Rictor expression, phosphorylation of AKT was statistically higher in DJ-1^−/−^ than in DJ-1^+/+^ iTregs (Fig. 7A,B). These results suggest that iTregs from DJ-1 deficient mice have higher AKT/Rictor signalling pathways which could lead to reduced iTregs development. We also characterised iTregs for TGF-β activated Smad2 protein levels and IL-2 receptor signalling, which could lead to Stat5 activation and to Foxp3 induction by binding to the transcription signalling site^10^. Indeed, phosphorylation of both signalling molecules was significantly less in DJ-1^−/−^ than in DJ-1^+/+^ iTregs (Fig. 7C,D). Collectively, TGF-β and IL-2R signalling was impaired in in DJ-1^−/−^ as compared with DJ-1^+/+^ iTregs leading to reduced Foxp3 induction.

### Lack of DJ-1 leads to enhanced ROS production and higher Sgk1 expression

**Figure 8:**
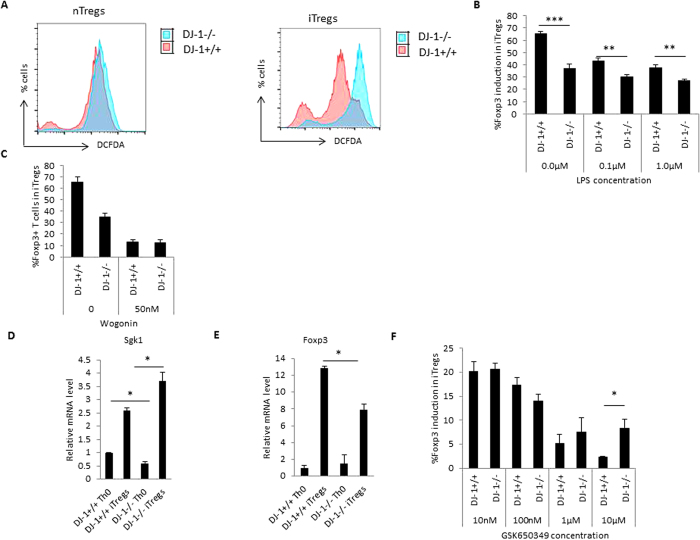
DJ-1 deficiency enhances the ROS production and affects Sgk1 expression in iTregs. (**A**) DJ1^−/−^ nTregs and iTregs have higher production of ROS measured by DCFDA dye compared with DJ-1^+/+^ mice. The pink histogram represents DJ-1^+/+^ and the overlay histogram in blue DJ-1^−/−^ iTregs. Data are derived from 3–4 independent experiments with similar observations. (**B**) Decreased iTregs induction after LPS treatment (0.1 μg/ml and 1.0 μg/ml) in DJ-1^−/−^ compared with DJ-1^+/+^ T cells. (**C**) Diminished Foxp3 induction after NFkB inhibitor wogonin (50 nM) treatment to DJ-1^−/−^ and DJ-1^+/+^ T cells. (**D**) Sgk1 expression was significantly lower in DJ-1^−/−^ Th0 cells compared with DJ-1^+/+^ mice (p = 0.02) however Sgk1 mRNA was upregulated in iTregs from both strains. DJ-1^−/−^ iTregs have statistically significant higher mRNA compared with DJ-1^+/+^ cells (p = 0.03). (**E**) Foxp3 mRNA was lower in DJ-1^−/−^ mice compared with DJ-1^+/+^ (p = 0.02). (**F**) Sgk1 inhibitor GSK650349 was employed in various doses as described in figure. At 10 μM concentrations, DJ-1^−/−^ T cells were subtle but significantly upregulate Foxp3 induction in developing iTregs. Data are shown mean ± SEM (n = 3–5 independent experiments and 6 biological replicate/group).

DJ-1 regulates the production of reactive oxygen species (ROS) and acts as an anti-oxidant^18^,^29^. We measured ROS production utilizing the 2, 7-dichlorodihydrofluorescein diacetate (DCFDA) dye in iTregs by flow cytometry. As illustrated in Fig. 8A, DJ-1^−/−^ iTregs produce more ROS than DJ-1^+/+^ iTregs. Further, we also found that isolated pTregs from DJ-1^−/−^ mice also produce a subtle but statistically significant amount of ROS (Fig. 8A). We previously showed that ROS can be induced by lipopolysaccharide (LPS) in dendritic cells (DCs) and that AKT1 deficient DCs produce less ROS compared to control DCs in presence of LPS^30^. Thus, we deduced that LPS treatment could enhance ROS and reduce the Foxp3 induction. We treated developing iTregs with LPS and found that LPS treatment could also inhibit the induction of iTregs in both wild type and DJ-1 deficient mice (Fig. 8B), in keeping with recently published findings^31^. It has previously been described that ROS down-regulates NFκB signalling in the nucleus^32^ leading to less Foxp3 induction. To test this hypothesis, we used 50 nM Wogonin (NFκB inhibitor)^33^, on developing iTregs, we confirmed that Wogonin treatment can reduce Foxp3 induction in DJ-1 deficient mice compared with control animals (Fig. 8C). Additionally, previous studies suggested that oxidative stress increases the expression of Sgk1 mRNA^34^,^35^. We thus explored Sgk1 mRNA levels in DJ-1^−/−^ mice using qRT-PCR. Sgk1 expression was significantly lower in Th0 cells from DJ-1^−/−^ mice however, contrasting results were observed in iTregs. mRNA expression was higher in DJ-1^−/−^ iTregs than in DJ-1^+/+^ iTregs (Fig. 8D,E). Thus, it appears that a higher expression of Sgk1 correlates with less Foxp3 expression in DJ-1 mice (Fig. 8F). To investigate this, we used a Sgk1 pharmacological inhibitor (10 nM–10 μM; GSK650349)^36^ and found that the Sgk1 inhibitor suppressed Foxp3 induction in DJ-1^−/−^ and DJ-1^+/+^ mice (Fig. 8E). The loss was not as high as in DJ-1^−/−^ mice but it did not enhance Foxp3 expression. Taken together, Sgk-1 and ROS could contribute to lower *in vitro* expression of iTregs in DJ-1 deficient mice.

### Inhibition of mTOR by rapamycin augments the iTregs induction

**Figure 9:**
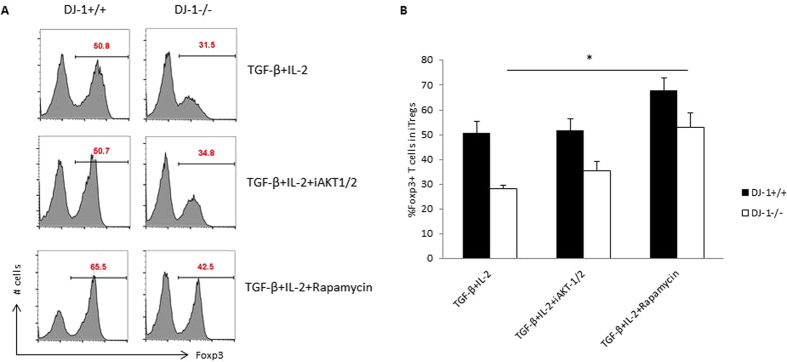
Inhibition of mTOR enhances the iTregs induction and appears to be DJ-1 independent. (**A**) Th0 cells were differentiated in the presence of TGF-β (2.5 ng/ml) and IL-2 (5.0 ng/ml) and AKT/mTOR pathways inhibitors such as AKT-1/2 (0.3 μM), Rapamycin (25 nM), as previously described concentrations^13^,^36^. Representative FACS plots show the %Foxp3 positive staining for various described treatments and (**B**) Foxp3 level was higher in Rapamycin treated cells in control DJ-1^+/+^ compared with DJ-1^−/−^ mice iTregs (p = 0.03). Data are shown mean ± SEM (n = 5 independent experiments).

As aforementioned, DJ-1-deficient mice have significantly higher AKT and Rictor expression in iTregs, we then explored whether attenuation of AKT or mTOR can lead to enhanced induction of Foxp3 in developing iTregs. We used pharmacological inhibitors for AKT (0.3 μM; iAKT1/2) and mTOR (25 nM; Rapamycin)^13^, to determine the pathway required for enhancing iTregs induction in the absence of DJ-1. The data in Fig. 9 point towards a general improvement of iTregs induction after mTOR inhibition and appears to be independent of DJ-1.

## Discussion

For effective immunotherapeutic treatment during infections, autoimmunity, neuro-inflammation and cancer, it is essential to understand Tregs development as these cells are pivotal in the maintenance of immune homeostasis^7^. The present observations shed light on a novel regulator of Tregs development, i.e. DJ-1 or PARK7.

DJ-1/PARK7 influences the clinical course of Parkinson´s disease (PD)^15^,^17^, which is characterized by progressive loss of dopaminergic neurons in the substantia nigra pars compacta and their terminal connections in the striatum^15^,^22^,^37^. Previous findings suggest that microglia neuro-inflammatory responses boost the neurodegeneration in PD^38^. Further studies suggested that the 1-methyl-4-phenyl-1,2,3,6-tetrahydropyridine (MPTP)-induced mouse model of PD can be protected by adoptive transfer of Tregs^39^. DJ-1 mRNA expression is enhanced in aged human pancreatic islets cells and reduced in type 2 diabetes mellitus (T2DM)^40^. In aged mice, there was significant increase in the nTregs number^41^ and Tregs deficiency heading to destruction of islet cells and immune-pathology^42^,^43^,^44^. This finding suggests that DJ-1 affects the development of Tregs, which could be instrumental in understanding the pathology of the neuro-immunology of PD and other autoimmune responses.

Various drugs such as PI3K inhibitors, AKT inhibitors and Rapamycin enhance whilst cyclosporine A can attenuate the development of iTregs^14^,^45^,^46^. Our data suggest that nTregs development was enhanced in the thymus as well as periphery and percentage of Tregs increases in DJ-1 deficient mice. However, total numbers of Tregs were almost equal in peripheral organs. These mice develop low levels of immunopathology when fed on high fat diet and CD4^+^ T cells were prone to differentiate into Th1 and Th2 cells^47^.

The *in vitro* experiments yielded the surprising result that DJ-1-deficient mice are unable to induce Foxp3 in developing iTregs. This is in contrast to the nTregs development and also the notion that DJ-1 deficiency enhances PTEN expression, thus attenuating the PI3K/AKT pathway and iTregs development. However, iTregs from DJ-1^−/−^ mice have less PTEN activity compared with control iTregs, a property in sharp contrast to observations in other cells types^11^,^20^. To resolve the disparity between the differential development of iTregs and nTregs, we measured cell proliferation and cell cycle assays and found that DJ-1-deficient iTregs are more proliferative and have poor in cell division compared with WT counterparts. Furthermore, iTregs from DJ-1 deficient mice are more prone to cell death. DJ-1 deficiency enhanced the death of developing iTregs but not of Th0 cells. Previous studies suggested that DJ-1 regulates the production of ROS and acts as an anti-oxidant^18^,^29^. Therefore, we also characterised the ROS production in developing iTregs and found that ROS production was severely enhanced in iTregs from DJ-1 deficient mice, which could lead to the enhanced death of these cells. However, previous studies suggested that ROS is upregulated in nTregs compared to Tcon cells and enhances the suppressive properties of Tregs^48^. In our experiments, it appears that enhanced ROS production lead to cell death and thus results in fewer iTregs.

Previous studies have suggested that ROS can enhance Rictor/mTOR signalling^49^. Enhanced Rictor leads to AKT phosphorylation which affects the Foxp3 stability. Thus, ROS could be a mechanism conferring of Foxp3 stability. TLR4 activation by LPS also affects Foxp3 in a dose dependant manner^31^. ROS has dual effects on NFκB, i.e. activation of cytoplasmic and inhibition of nuclear protein^32^. According to our observations NFκB inhibition leads to further iTregs reduction and is consistent with previous findings^50^. In addition, a recent study showed that ROS production is reduced in AKT1 deficient dendritic cells^30^. As AKT deficiency leads to higher Foxp3 induction^14^, it could affect Foxp3 stability in DJ-1 deficient mice. Our data also revealed less Smad2 phosphorylation in DJ-1 deficient iTregs compared with littermate control iTregs, an observation suggesting that DJ-1 is also involved in IL-2 receptor signalling. DJ-1^−/−^ iTregs also shows less pStat5 activity compared with DJ-1^+/+^ iTregs. It appears that *in vitro* DJ-1^−/−^ iTregs are less sensitive to TGF-β/IL-2 signalling due to less pSmad2/pStat5 phosphorylation. Furthermore, we speculate that TCR activation by anti-CD3/anti-CD28 leads to the activation of the AKT/mTOR pathway. It appears to be more dominating than TGF-β/IL-2 signalling in DJ-1^−/−^ developing iTregs and TGF-β/IL-2 signalling communication may be outcompeted during this process due to highly proliferative nature of Foxp3^-^ T cells and thus, could lead to lower Foxp3 induction. Nonetheless, an actual contribution of both signalling pathways will require further research. In summary, DJ-1 affects the *in vitro* development of iTregs and switching the signalling from *in vivo* developed nTregs. However, further studies are warranted to find the exact molecular mechanism for *ex vivo* isolated nTregs and iTregs.

Enhanced ROS production may increase Sgk1 expression^34^,^35^. Sgk1 could in turn lead to the phosphorylation of FoxO1/3a protein which could destabilise the Foxp3 protein^46^,^51^. However, inhibition of mTOR/Sgk1 is required for the development of iTregs in DJ-1-deficient mice but not in wild type mice. Therefore, it appears that a balanced Sgk1 activity is required for iTregs development. If the expression level reaches a threshold, it may lead to the induction of pathogenic Th-17 cells^52^,^53^. DJ-1-deficient mice showed a low-level inflammatory response in the high-fat diet-induced obesity model^47^. In contrast infiltration of T cells occurs in multiple sclerosis and PD, and levels of IL-1β, IL-6 and TNF-α are elevated in the cerebrospinal fluid of PD patients^54^. A recent study suggested that DJ-1 deficient CD4^+^ T cells are also able to differentiate into more Th1 and Th17 phenotypes in an atherosclerosis model^23^. Therefore, during inflammation in DJ-1^−/−^ mice iTregs presumably fail to develop adequately thus influencing disease severity.

In conclusion, our study identified a novel role of DJ-1 in differential regulation of nTregs and iTregs *via* regulating mTOR/Sgk1 axis as well through ROS/NFκB signalling pathways. DJ-1 thus participates in the regulation of the immune response.

## Materials and Methods

### Mice

DJ-1^+/+^ and DJ-1^−/−^ (10–12 week-old) mice were used for the experiments as described earlier^18^ and kept under standard conditions. All experiments were performed according to the EU Animals Scientific Procedures Act and the German law for the welfare of animals. The procedures were approved by the authorities of the state of Baden-Württemberg.

### CD4^+^ T cell purification

To perform the iTreg induction experiments, naïve CD4^+^CD25^-^ T cells were isolated from above 10–12 week old female mice using magnetic bead selection. To isolate CD4^+^ T cells, spleen and lymph nodes (inguinal, axillary, brachial, mediastinal, superficial cervical, mesenteric) were collected from the mice and macerated using a syringe plunger. The cell suspension was centrifuged at 600 × *g* at 4 °C for 5 minutes and the cell pellet was treated with RBC lysis buffer for 1 minute and then washed for three times with 10% RPMI1640 medium (Invitrogen, Germany). After washing, cells were kept on a roller at 4 °C (cold room) for 30 minutes in the presence of 50 μl/mouse antibody mix containing anti-CD8, anti-MHC II, anti-CD11b, anti-CD16/32, anti-CD45R, and Ter-119 (Dynabeads® Untouched™ Mouse CD4 cells kit, Invitrogen, Germany). Cells were washed after antibody incubation andcounted and added 1:1 (cells to beads ratio) Dynabeads in 1.0 ml media/mouse and incubated at 4 °C (cold room) for 30 minutes on a roller to deplete the CD8^+^ T cells, B cells, NK cells, monocytes/macrophages, dendritic cells, erythrocytes and granulocytes. Using the Magnet stand (DynaMag™ 5 Magnet), all these Dyanbeads-bound cells were captured and the supernatant containing CD4^+^ T cells was collected. This process is called negative selection of CD4^+^ T cells. Using MACS separation columns CD4^+^CD25*+* T cells were positively selected and remaining cells were CD4^+^CD25^-^ T cells^55^.

### iTregs differentiation

CD4^+^CD25^−^ T cells were activated in the presence of plate-bound anti-CD3/anti-CD28 antibodies (eBiosciences, UK) with a ratio of 1:2::anti-CD3:anti-CD28 (1.0 μg/ml anti-CD3: 2.0 μg/ml anti-CD28) for iTregs. Briefly, naïve T cells were differentiated into iTregs using 5.0 ng/ml recombinant-TGF-β, 10.0 ng/ml recombinant-IL-2 (eBiosciences, Germany), cultured for 3–4 days in RPMI1640 medium (Invitrogen) supplemented with FBS, Penicillin/Steptomycin, L-glutamine, β-Mercapto ethanol^55^. Cells were harvested at day 3–4 and used for intracellular staining using flow cytometry, q-RT-PCR and immune-blotting experiments.

### Flow cytometry

Thymocytes and splenocytes from wild type and DJ-1-deficient mice were characterised by using surface and intracellular staining with relevant antibodies. In brief, thymocytes and splenocytes were collected and used for surface staining for anti-CD4, anti-CD8a, anti-CD25, anti-CD62L, anti-CD44 (eBioscience, Germany) or other antibodies depending on the experiment and washed with PBS. Cells were fixed with Foxp3 fixation/permeabilization buffer (eBioscience, Germany) for intracellular staining and incubated for 30 minutes. After incubation, cells were washed with 1x permeabilization buffer, exposed to added intracellular monoclonal antibodies for Foxp3 and incubated for additional 30 minutes. Cells were washed again with permeabilization buffer and PBS was added to acquire the cells on a flow cytometer (FACS-calibur from Becton Dickinson; Heidelberg, Germany).

### Cell cycle analysis

Activated Th0 and iTregs were washed 1x with PBS at 600xg for 5 minutes at RT. After discarding the supernatant, 1 ml of −20 °C cold ethanol:PBS mix (3:1) was added during swirl mixing of the cells. The preparation was kept at −20 °C overnight to maximum 4 days depending on the experiment. After incubation at −20 °C, cells were washed 1× with PBS again and 250 μl of PI mix were added [50 μg/ml PI (Sigma, Germany) and 100 μg/ml RNase A (Qiagen, Germany)]. The cells were kept for 30 minutes at RT. Then, the cells were acquired by flow cytometry for cell cycle and death analysis.

### CFSE staining and cell proliferation

CD4^+^ T cells (5 × 10^6^) were washed 1× with PBS (Sigma, Germany) and stained with 2 μM CFSE (eBioscience, Germany) for 15 minutes at RT in the dark and washed 2× with RPMI-1640 medium as described earlier^55^. Stained cells were cultured for 3 days and after 3 days of culture; cells were stained with Foxp3 antibody and acquired on flow cytometry. For suppression assay conventional T cells and nTregs (DJ-1^+/+^ and DJ-1^−/−^) were added into 1:1 ratio and run on the flow cytometry to measure the proliferation of conventional cells as described previously^55^.

### Immunoblotting

Naïve T cells (1 × 10^6^ cells) from DJ-1^+/+^ and DJ-1^−/−^ were activated in the presence of anti-CD3::anti-CD28 (1:2) and differentiated into iTregs in presence of TGF-β and IL-2 and after 3 days of culture, cells were lysed using Lamelli buffer and subjected to immunoblotting. Differentiated iTregs were activated for 30 minutes with respective conditions and then washed once with PBS and added the equal amount of H_2_O and 2X Lammelli’s Buffer for cell lysis and proteins was denatured at 95 °C for 5 minutes and stored at −20 °C. Sample proteins were loaded on 8% or 10% gel depending on proteins size and run for 80–120 mV for 90–100 minutes. Proteins were electrotransferred onto PVDF membrane (GE healthcare, USA). Membranes were probed with the indicated primary antibodies (Pten, pAktS473, mTOR, Rictor, pSmad2, pStat5, DJ-1 and GAPDH; Cell Signalling, USA) followed by HRP-conjugated secondary antibodies (Cell Signalling, USA). Membranes were washed and visualized with enhanced chemiluminescence detection system (ECL; peqLab, Germany).

### q-RT-PCR

Total mRNA was isolated from Th0 and iTreg cells using the mRNAeasy isolation kit (Qiagen, Germany) as described by the manufacturer. 1.0 μg mRNA was converted into cDNA using the Superscript III cDNA synthesis kit (Invitrogen, Germany). Briefly, in 10.0 μl reactions, 10.0 ng cDNA, 2X SYBR green Master-mix (KAPA SYBR green, peqLab, Germany) and 250 nM primers were used for q-RT-PCR reactions. q-RT-PCR and data analysis were performed as described previously^56^ for Foxp3 (F primer: 5′- GGTACACCCAGGAAAGACAG-3′ and R primer: 5′-ATCCAGGAGATGATCTGCTTG-3′), Sgk-1 (F primer: 5′-TGAAACAGAGAAGGATGGGC-3′ and R primer: 5′-GAACTTCAGCGTGTTTGCAT-3′) using universal cycling conditions (95 °C for 10 minutes, 95 °C for 15 seconds and 60 °C for 1 minute for 40 cycles followed by melting curve analysis). All the primers were purchased from Sigma, Germany.

### ROS production

ROS production in iTregs was measured by 2′,7′-dichlorodihydrofluorescein diacetate (DCFDA). Briefly, 1 × 10^6^ cells were taken in a 24 well plate and DCFDA (Sigma, Germany) was added to the cell suspension at a final concentration of 10 μM. After 30 minutes of incubation in the dark at RT, cells were centrifuged and the pellet was washed twice with 1x PBS. The pellet was then resuspended in FACS buffer and the fluorescence was analysed with a flow cytometer. DCFDA fluorescence intensity was measured in FL-1 with an excitation wavelength of 488 nm and an emission wavelength of 530 nm.

### Statistics

Figures were made in Excel Microsoft office software. Arithmetic mean values are presented ± standard error of mean (SEM) and n represents the number of independent biological experiments. GraphPad Prism and Excel were used for statistical analyses. Student’s unpaired t-test was used for significance. P values of equal or less than 0.05 were considered significant, **p≤0.005, ***p≤0.0005 and ****p≤0.00005.

## Additional Information

**How to cite this article**: Singh, Y. *et al.* Differential effect of DJ-1/PARK7 on development of natural and induced regulatory T cells. *Sci. Rep.* **5**, 17723; doi: 10.1038/srep17723 (2015).

## Supplementary Material

Supplementary Information
