# Supplementary material for: Differential effect of DJ-1/PARK7 on development of natural and induced regulatory T cells
Source: Sci Rep. 2015 Dec 4;5:17723. doi: 10.1038/srep17723 (PMC4669505; doi:10.1038/srep17723)
Supplement: Supplementary Information [file srep17723-s1.pdf]

Differential effect of DJ-1/Park-7 on development of natural and induced regulatory T cells

Yogesh Singh[1*], Chen Hong[1], Yuetao Zhou[1], Michael Föller[1,2], Tak W. Mak[2], Madhuri S Salker[1], Florian Lang[1*]

[1]Department of Physiology I, Eberhard-Karls-University of Tübingen, Tübingen, 72076, Germany
[2]Campbell Family Institute for Breast Cancer Research, Ontario Cancer Institute, UHN, 620 University Ave Toronto, M5G 2C1, Canada

[*]E-Mail: ysinghbt@gmail.com or florian.lang@uni-tuebingen.de

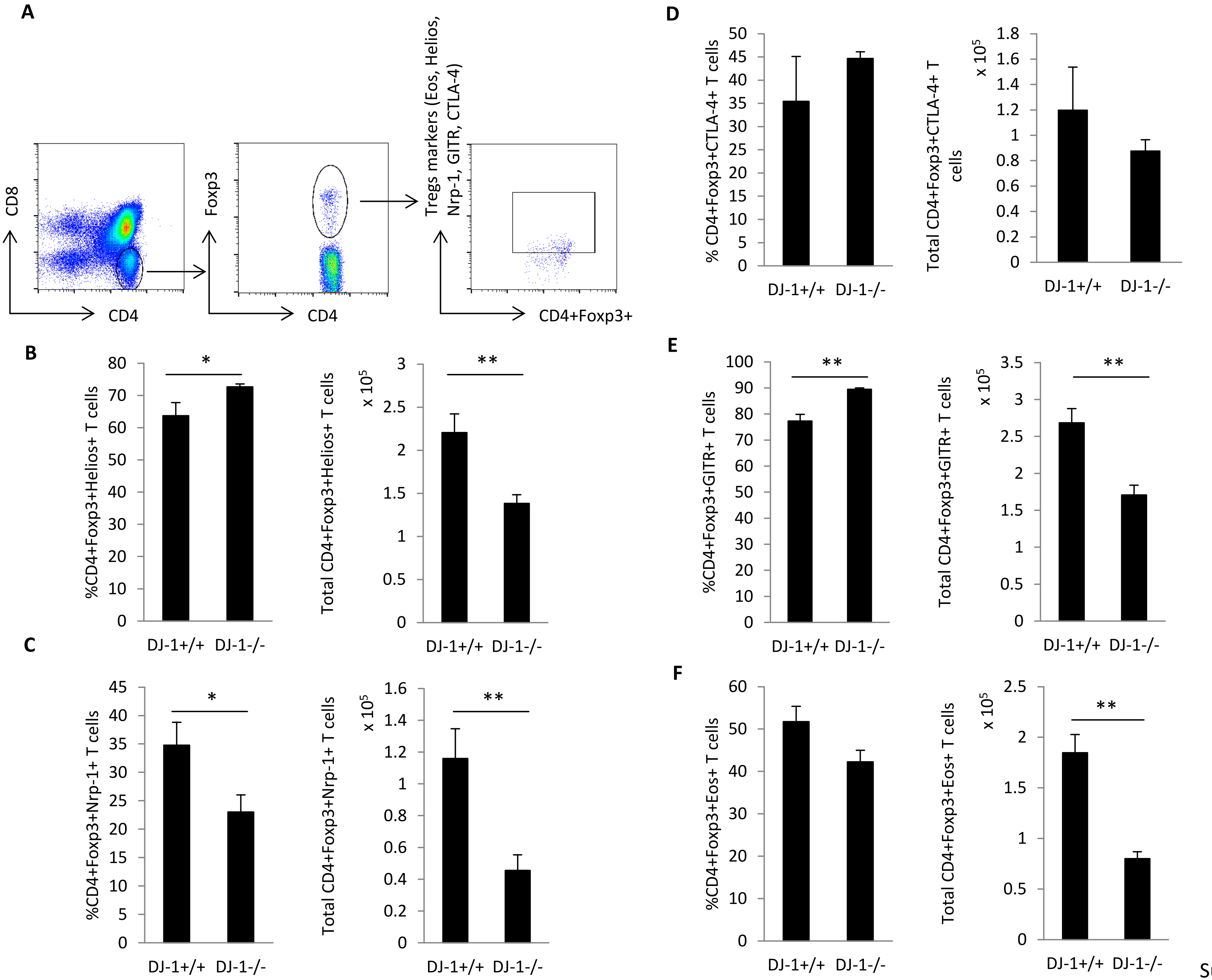

A
CD8
CD4
Foxp3
CD4
Tregs markers (Eos, Helios, Nrp-1, GITR, CTLA-4)
CD4+Foxp3+
B
%CD4+Foxp3+Helios+ T cells
Total CD4+Foxp3+Helios+ T cells
x 10^5
C
%CD4+Foxp3+Nrp-1+ T cells
Total CD4+Foxp3+Nrp-1+ T cells
D
% CD4+Foxp3+CTLA-4+ T cells
Total CD4+Foxp3+CTLA-4+ T cells
E
%CD4+Foxp3+GITR+ T cells
Total CD4+Foxp3+GITR+ T cells
F
%CD4+Foxp3+Eos+ T cells
Total CD4+Foxp3+Eos+ T cells
DJ-1+/+
DJ-1-/-

Suppl. Fig. 1

Suppl. Fig.1 Characterisation of thymic Tregs in DJ-1 deficient mice: Statistical summary
(A-F) DJ-$1^{+/+}$ and DJ-$1^{-/-}$ thymocytes were stained with anti-CD4, anti-CD8, anti-Foxp3 mAbs and characterised for Eos, Helios, CTLA-4, GITR and Nrp-1 expression. Total thymic cells were gated for $CD4^{+}$ marker and these $CD4^{+}$ thymocytes were gated again for $Foxp3^{+}$ marker, these $CD4^{+}Foxp3^{+}$ gated population were characterised for Tregs markers (Helios, Nrp-1, GITR, CTLA-4, and Eos). Bar diagrams represent Mean ± SEM of all FACS plot as we as well total number of thymocytes (corresponding Fig.2). n=2 independent experiments and 5-6 biological replicates/group). Student's unpaired t-test was used for significance. P values of equal or less than 0.05 were considered significant, p≤0.05*, p≤0.01** and p≤0.001***.

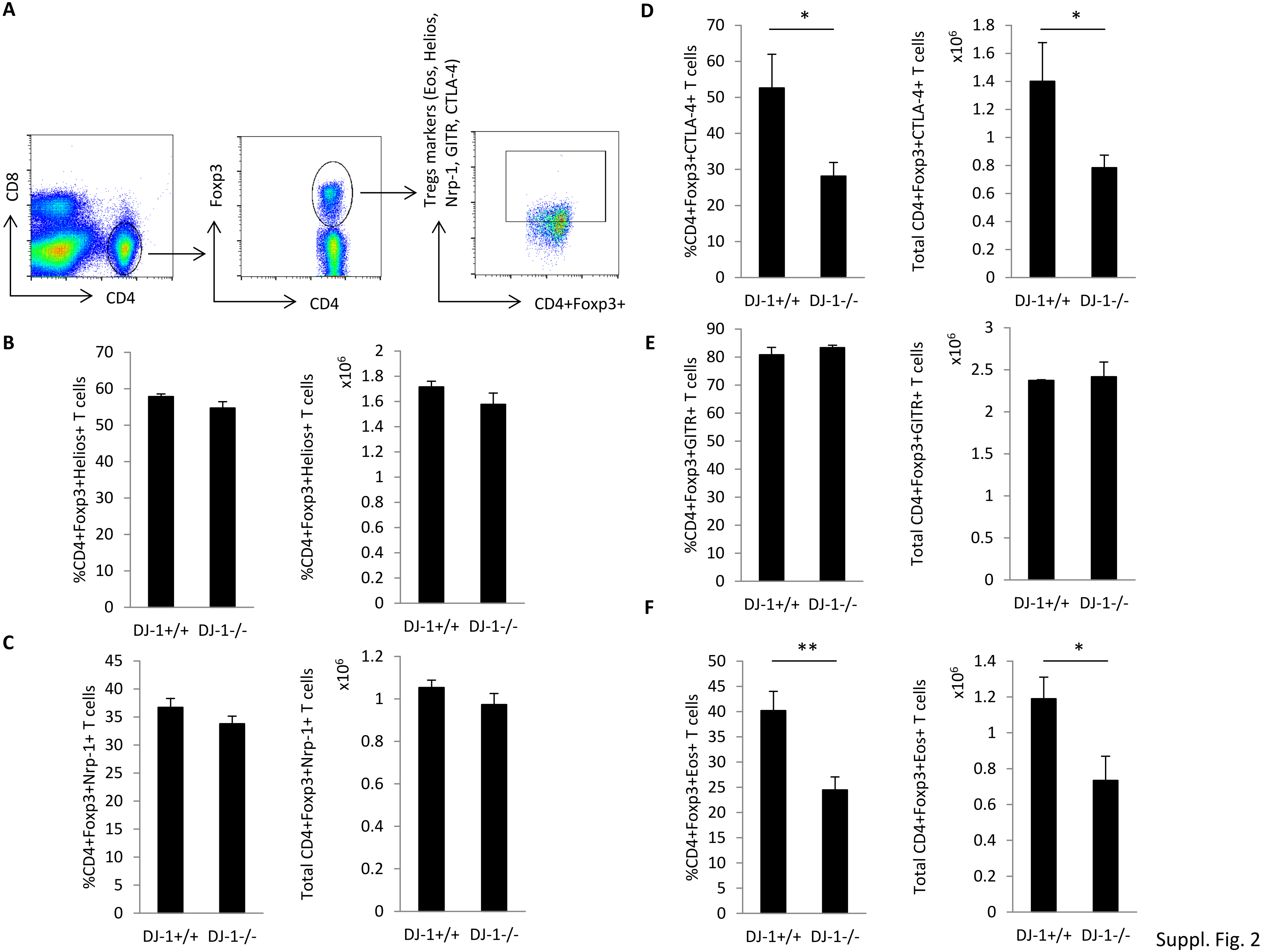
A
CD8
CD4
Foxp3
CD4
Tregs markers (Eos, Helios, Nrp-1, GITR, CTLA-4)
CD4+Foxp3+
B
%CD4+Foxp3+Helios+ T cells
DJ-1+/+
DJ-1-/-
x10^6
C
%CD4+Foxp3+Nrp-1+ T cells
Total CD4+Foxp3+Nrp-1+ T cells
D
%CD4+Foxp3+CTLA-4+ T cells
Total CD4+Foxp3+CTLA-4+ T cells
*
E
%CD4+Foxp3+GITR+ T cells
Total CD4+Foxp3+GITR+ T cells
F
%CD4+Foxp3+Eos+ T cells
Total CD4+Foxp3+Eos+ T cells
**

Suppl. Fig. 2

Suppl. Fig.2 Characterisation of nTregs in DJ-1 deficient mice from iTregs: Statistical summary

(A-F) DJ-$1^{+/+}$ and DJ-$1^{-/-}$ splenocytes were stained with anti-CD4 and anti-CD8 mAbs and characterised for Foxp3, Eos, Helios, CTLA-4, GITR and Nrp-1 expression. Bar diagrams represent Mean ± SEM of all FACS plot as well as total $CD4^{+}Foxp3^{+}$ T cells shown in Fig.4 and represent the staining for $CD4^{+}$ T cells gated for $CD4^{+}Foxp3^{+}$ *versus* Helios, Nrp-1, CTLA-4, GITR and Eos. (n = 2 independent experiments and 5-6 biological replicates/group). Student's unpaired t-test was used for significance. P values of equal or less than 0.05 were considered significant, $p \leq 0.05$*, $p \leq 0.01$** and $p \leq 0.001$***.

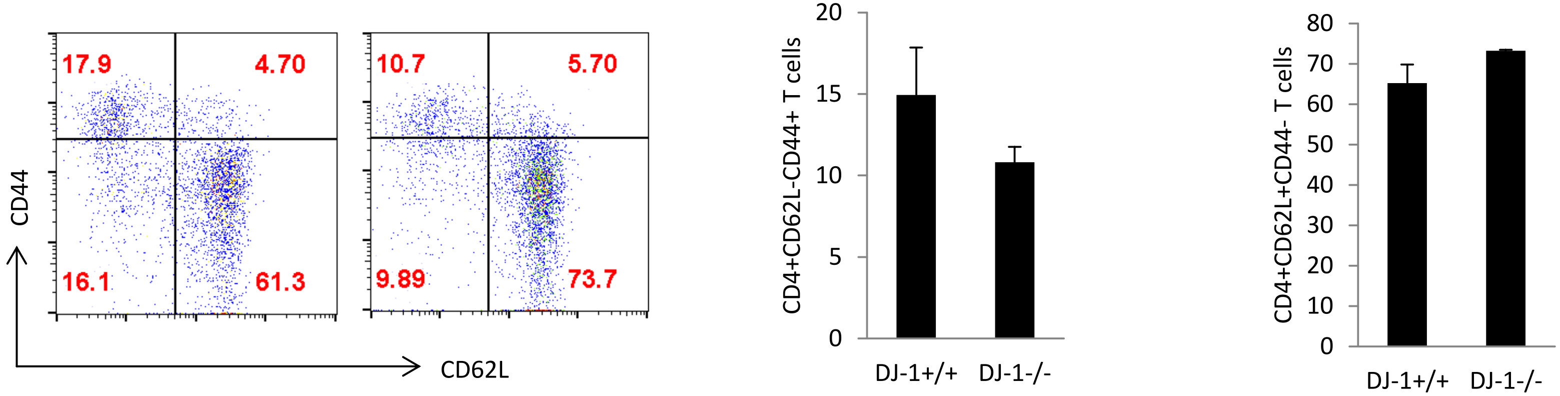
17.9
4.70
16.1
61.3
10.7
5.70
9.89
73.7
CD44
CD62L
CD4+CD62L-CD44+ T cells
20
15
10
5
0
DJ-1+/+
DJ-1-/-
CD4+CD62L+CD44- T cells
80
70
60
50
40
30
20
10
0
DJ-1+/+
DJ-1-/-

Suppl. Fig. 3

Suppl. Fig.3 Characterisation of naïve and memory T cells from DJ-1 deficient mice
$CD4^+$ T cells were characterised for naïve and memory T cells markers and based on CD44 and CD62L expression, no significant difference was observed in between DJ-$1^{+/+}$ and DJ-$1^{-/-}$ mice. Left hand side shows the representative FACS plots for CD44 and CD62L staining gated on $CD4^+$ T cells and right hand side shows mean ± SEM (n = 3 independent experiments) for CD44 and CD62L expression.

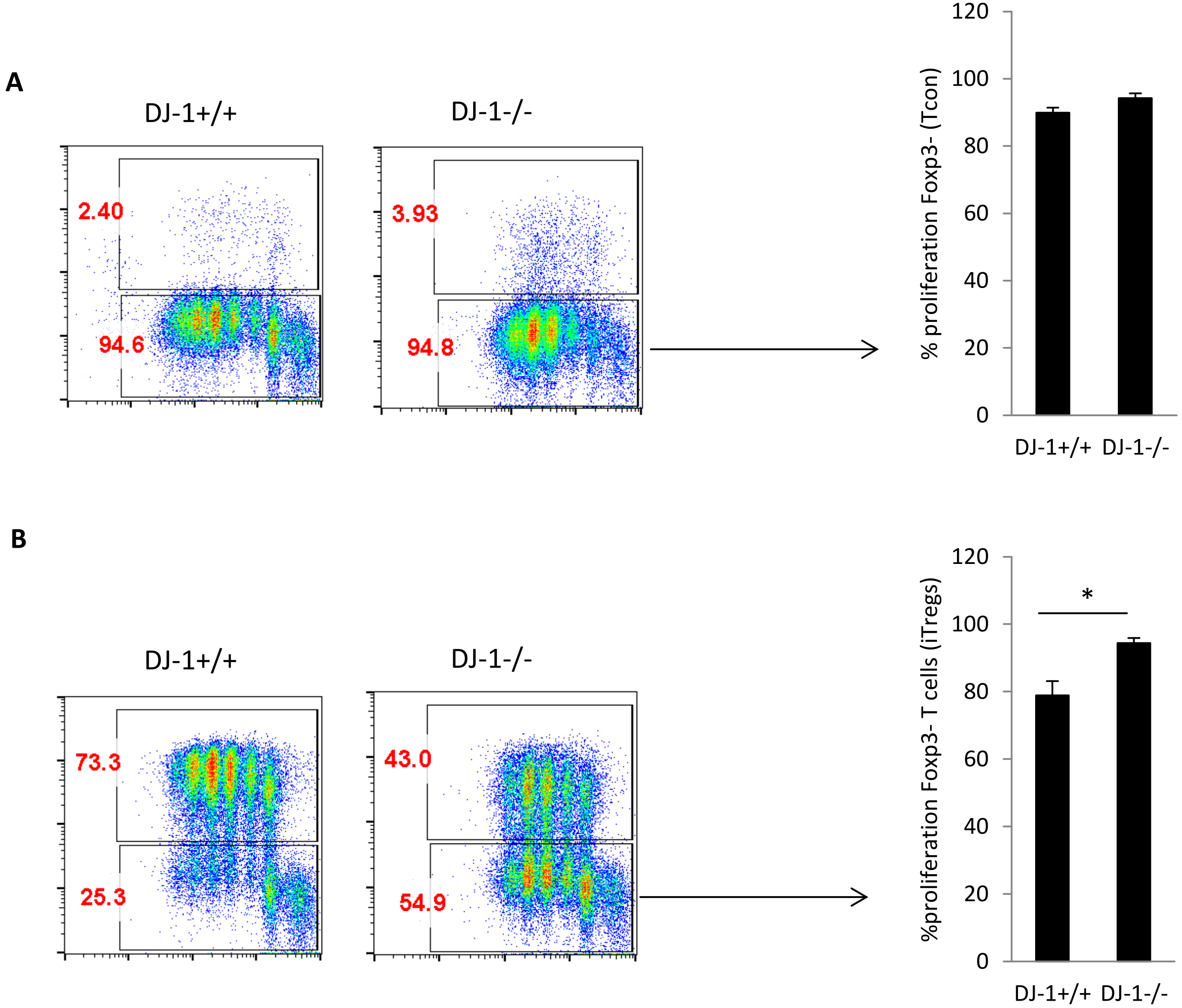
A
DJ-1+/+
DJ-1-/-
2.40
94.6
3.93
94.8
% proliferation Foxp3- (Tcon)
120
100
80
60
40
20
0
DJ-1+/+ DJ-1-/-
B
DJ-1+/+
DJ-1-/-
73.3
25.3
43.0
54.9
%proliferation Foxp3- T cells (iTregs)
*
120
100
80
60
40
20
0
DJ-1+/+ DJ-1-/-

Suppl. Fig. 4

Suppl. Fig. 4 DJ-1 deficient Foxp3$^-$iTregs are more proliferative in nature compared to wild type controls mice

Purified CD4$^+$ T cells were stained with the cell proliferation dye CFSE (2 µM) and cultured for 3 days with TGF-β (5.0 ng/ml) and IL-2 (10.0ng/ml) (iTregs) and without both cytokines (Th0). After 3 days of culture cells were stained with a Foxp3 antibody and acquired by flow cytometry and measured the proliferation of Foxp3$^+$ and Foxp3$^-$ iTregs as well as Th0. Flow data suggested that Foxp3$^-$ DJ-1$^{-/-}$ mice have significantly higher proliferation in iTregs compared with DJ-1$^{+/+}$ mice ($p=0.027$), whereas no difference was noticed in Foxp3$^-$Th0 cells in between both strains of mice. Left hand side shows the FACS plots whereas right hand side shows the mean ± SEM ($n = 3$ independent experiments) proliferation for Th0 cells and iTregs respectively.
